# Divergent Evolutionary Pressures Shape Olfactory Sensitivity of the Maxillary Palps in Tephritidae Fruit Flies

**DOI:** 10.1002/ece3.72261

**Published:** 2025-10-14

**Authors:** Chaymae Fennine, Sebastian Larsson Herrera, Tibebe Dejene Biasazin, Wittko Francke, Sergio Angeli, Teun Dekker

**Affiliations:** ^1^ Chemical Ecology Unit, Department of Plant Protection Biology Swedish University of Agricultural Sciences Alnarp Sweden; ^2^ Faculty of Agricultural, Environmental and Food Sciences Free University of Bozen‐Bolzano Bolzano Italy; ^3^ National Species Management Wageningen the Netherlands; ^4^ Institute of Organic Chemistry University of Hamburg Hamburg Germany

**Keywords:** GC‐EPD, maxillary palps, olfactory sensitivity, tephritids

## Abstract

Olfaction is a rapidly evolving sense. Given its diverse functions, from finding ecological niches to selection of mates, we hypothesized that olfaction is subjected to divergent evolutionary pressures. We compared the olfactory sensitivity of five species of Tephritidae fruit flies to two broad classes of volatiles: general niche‐related volatiles (food and fruit odors) and volatiles used in sexual communication (pheromones and “parapheromones”). We then analyzed whether the differential sensitivities across species harbor “signals” of such contrasting evolutionary pressures. As recent studies highlight the maxillary palps as key auxiliary olfactory organs for detecting both classes of volatiles, we focused our sensory analysis on this auxiliary olfactory organ. Using gas chromatography coupled to electropalpographic detection (GC‐EPD), we recorded sensory responses from five species with a diverse phylogenetic and ecological separation. Detection overlapped considerably across taxa; however, the maxillary palp exhibited distinct sex and clade‐specific patterns in sensitivity to pheromones and parapheromones. Cluster analysis of sensitivities to (para)pheromones aligned strongly with the species' phylogeny. In contrast, cluster analysis of sensitivities to general food and fruit odors clustered separately and showed a strong correlation with ecological niche rather than phylogeny. Clearly, the selection pressures that shape the evolutionary direction of olfactory sensitivity to (para)pheromones and niche‐related odors are diametrical opposites, reminiscent of stabilizing versus directional selection. Understanding the detection and evolution of distinct volatile classes provides valuable insights into the evolutionary ecology of olfaction, studies on olfactory receptors, and sensory and preference coding, and supports the rational development of novel lures to manage these pest insects.

## Graphical abstract

Tephritids maxillary palps are particularly sensitive to pheromones and male lures, and these olfactory sensitivities are shaped by evolutionary forces.

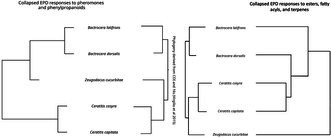

## 1 Introduction

Insets, which account for over 80% of all described species, represent the most diverse group of organisms (Stork 2018). Their short generation time and small size allow insects to rapidly invade new niches. Their evolutionary adaptability is paralleled by an equally rapidly evolving sense of smell, which detects key environmental cues, such as food sources and potential mates. This is for instance reflected at the protein level in olfactory receptors (ORs), with radiation events generally reflected in OR diversification (Missbach et al. 2014). However, whether the diversification of OR sequences equals functional radiation is largely unknown, partially due to the backlog of functional characterization of ORs. Similarly, it is largely unknown whether the evolutionary pressures acting on ORs are the same, irrespective of their function. It is, however, conceivable that the selection pressures acting on the detection of for example, food or oviposition site volatiles differ from the detection of sexual communication signals. We addressed this question by dissecting the odor sensitivity of the maxillary palps of tephritid fruit flies, which is sensitive to both fruit and food odors on the one hand, and sexual signals on the other.

In insects, the antennae serve as the primary olfactory organs, but many species also possess auxiliary olfactory structures such as the maxillary palps. Olfactory sensilla can also occur in other anatomical regions, including the ovipositor of *Manduca sexta* , where they detect odorants and may play a role in reproductive behaviors (Klinner et al. 2016), and the labial pit of certain adult Lepidoptera, which houses CO_2_‐sensitive sensilla (Kent et al. 1986). The maxillary palps, located on the proximal part of the mouthparts, the labium, house a limited number of sensory neuron types compared to the antennae. In *Drosophila*, the maxillary palps contain only one morphological type of olfactory sensillum, the basiconic sensillum, with three subtypes, each harboring two olfactory sensory neuron (OSN) types (de Bruyne et al. 1999; Dweck et al. 2016). Similarly, in Tephritidae, the maxillary palps express three sensilla basiconica subtypes, each harboring two sensory neurons (Larsson‐Herrera et al. 2024). Due to the proximity of feeding substrates, maxillary palps have been hypothesized to play a disproportionate role in detecting food‐related odors, complementing the broader olfactory functions of the antennae. Whereas this was not evident in *Drosophila*, in Tephritidae, the palps exhibited a preferential sensitivity to food odors over fruit odors (Larsson‐Herrera et al. 2024).

In addition to being sensitive to fruit and food odors, the palps in Tephritidae are also sensitive to spiroacetals (Noushini, Park, et al. 2020; Noushini, Perez, et al. 2020; Noushini et al. 2021). This group of compounds is produced in the rectal gland of *Bactrocera* species and, as they mediate sexual behaviors, are recognized pheromones of Tephritidae (Haniotakis 1974; Baker et al. 1980; Zhang et al. 1997; Booth et al. 2007; Noushini, Park, et al. 2020; Noushini, Perez, et al. 2020; Noushini et al. 2021). They have, however, not been reported from *Ceratitis* spp. Whereas spiroacetals are detected by the maxillary palps of *Bactrocera bryoniae, B* *. kraussi* , *B. frauenfeldi*, and *B. oleae* (Noushini, Park, et al. 2020; Noushini, Perez, et al. 2020; Noushini et al. 2021), little is known about the detection in other species. Given the variety of spiroacetals and differences between sexes (Booth et al. 2009), comparative sensory studies would be helpful in understanding how these pheromones are detected, how their detection has evolved, and how olfactory input translates into behavior.

Another group of compounds to which the palps are sensitive is phenylpropanoids (Chieng et al. 2018; Park et al. 2018; Verschut et al. 2018). Phenylpropanoids are of plant origin and typically attract males. Given their strong and sex‐biased attractiveness, they are often referred to as parapheromones. Pheromones and parapheromones are extensively used in the monitoring and control of several Tephritidae species. Methyl eugenol (ME) is used for mass trapping, attract‐and‐kill, and monitoring of species; for example, *B. dorsalis* , raspberry ketone (RK), and its synthetic analog cuelure (CL) are employed for monitoring species, such as *Z. cucurbitae* (Clarke 2019; Biasazin et al. 2021). *Ceratitis* species do not appear to be attracted to phenylpropanoids, but two other male lures, terpinyl acetate and the synthetic chlorinated trimedlure, fulfill a similar role for the monitoring and control of *C. cosyra* and *C. capitata* , respectively. Beyond detection, the palps are also essential for the orientation toward parapheromones in several tephritid species, as evidenced by ablation experiments in *B. dorsalis* (Chieng et al. 2018).

In this study, we evaluated the responses of the maxillary palps of males and females from phylogenetically and ecologically diverse tephritid species using coupled gas chromatography electropalpographic detection (GC‐EPD). We investigated the olfactory sensitivities of the maxillary palps in multiple tephritid species. These included *B. dorsalis* (Hendel), a polyphagous and severe horticultural pest that releases the spiroacetal (2*E*,8*E*)‐2‐ethyl‐8me‐1,7‐dioxaspiro[5.5]undecane (Perkins et al. 1990) and is attracted to methyl eugenol (Kawano et al. 1968); *Bactrocera latifrons* (Hendel), a solanaceous specialist whose males are attracted to isophorone (Ishida et al. 2008) and produces the same spiroacetal as *B. dorsalis*, as well as (2S,6R,8S)‐2,8‐dimethyl‐1,7‐dioxaspiro[5.5]undecane (Zhang et al. 1997); and the cucurbit specialist *Zeugodacus cucurbitae* (Coquillet), which is attracted to RK/CL (Kawashita et al. 2004) and is a known producer of spiroacetal (2*E*,8*E*)/(2*Z*,8*E*)‐2‐ethyl‐8me‐1,7‐dioxaspiro[5.5]undecane (Baker and Bacon 1985). We also included two polyphagous species of *Ceratitis*: *C. capitata* (Wiedemann) and *C. cosyra* (Walker), which are attracted to trimedlure (Beroza et al. 1961) and terpinyl acetate (White and Elson‐Harris 1992), respectively. However, neither of these *Ceratitis* species have been observed to produce spiroacetals. The list of stimuli further included compounds such as phenylpropanoids, esters, pyrazines, and phenols, which were selected based on literature (Biasazin et al. 2018; Chieng et al. 2018; Segura et al. 2018; Ono et al. 2021; Larsson‐Herrera et al. 2024). Using synthetic compounds in conjunction with GC‐EPD also removes false positives from impurities that may arise when employing non‐GC approaches such as SSR and EAG/EPG with puffing (Schorkopf et al. 2019). The data provide insights into palpal detection and its evolutionary role in mediating sexually divergent behavioral responses to specific compounds. Finally, since these compounds are important in pest control, understanding how they are detected may provide valuable insights for their use in pest management.

## 2 Material and Methods

### 2.1 Insects

Lab colonies of the fly species were established from pupae obtained from the International Center of Insect Physiology and Ecology (*icipe*, Kenya; *B. dorsalis* , *B. latifrons* , *C. capitata* , *C. cosyra* ), and the International Atomic Energy Agency (IAEA, Vienna, Austria; *Z. cucurbitae*). Emerging adult flies were kept in polyester netting Bugdorm cages (325 × 325 × 325 mm^3^) under controlled conditions (25°C, 60% ± 5% RH and 12:12 LD), and provided with food (sugar and baker's yeast (Jästbolaget AB, Sollentuna, Sweden) mix, 3:1) and water (wet cotton).

### 2.2 Chemicals

Authentic chemical standards (> 95% purity) of compounds are summarized in Table S1. Most of the compounds were purchased from Sigma, Aldrich, St. Louis, MO, USA, whereas others were from various sources and are available at the local chemical library of the Swedish University of Agricultural Sciences (SLU), Alnarp. The spiroacetals (except olean, provided by ISCA Technologies) were synthesized by Professor Wittcko Francke (Table 1).

**TABLE 1:** Spiroacetals and male lures tested on the maxillary palps of five tephritids ( *Bactrocera latifrons* , *Bactrocera dorsalis* , *Zeugodacus cucurbitae*, *Ceratitis capitata* , and *Ceratitis cosyra* ).

Class	Trivial name	IUPAC name	Structure
Spiroacetals	Spiro A	(2R)‐2‐methyl‐1,7‐dioxaspiro[5.5]undecane	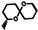
	Spiro B	2‐ethyl‐1,7‐dioxaspiro[5.5] undecane (racemate)	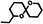
	Spiro C	(2S,6R,8S)‐2,8‐dimethyl‐1,7‐dioxaspiro[5.5] undecane	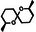
	Spiro D	(2S,6S,8R)‐2,8‐dimethyl‐1,7‐dioxaspiro[5.5] undecane	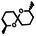
	Spiro E	2‐ethyl‐8‐methyl‐1,7‐dioxaspiro[5.5]undecane (racemate, fraction 1)	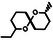
	Spiro F	2‐ethyl‐8‐methyl‐1,7‐dioxaspiro[5.5]undecane (racemate, fraction 2)	
	Spiro G	2‐ethyl‐8‐methyl‐1,7‐dioxaspiro[5.5]undecane (racemate, fraction 3)	
	Olean	1,7‐dioxaspiro[5.5] undecane (racemate)	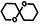
Phenylpropanoids	Methyl eugenol	1,2‐dimethoxy‐4‐prop‐2‐enylbenzene	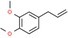
	Raspberry ketone	4‐(4‐hydroxyphenyl) butan‐2‐one	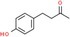
	Cuelure	[4‐(3‐oxobutyl)phenyl] acetate	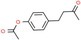
	Zingerone	4‐(4‐hydroxy‐3‐methoxyphenyl)butan‐2‐one	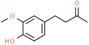
Terpenoids	Trimedlure	tert‐butyl 4‐chloro‐2‐methylcyclohexane‐1‐carboxylate	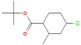
	Terpinyl acetate	2‐(4‐methylcyclohex‐3‐en‐1‐yl)propan‐2‐yl acetate	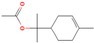

### 2.3 Synthetic Blends

Three blends were constructed of a total of 40 synthetic compounds (Table 1). Some of the compounds, such as spiroacetals, male lures, and others, were selected based on literature data from tephritids and drosophilids. Others were either known from our own unpublished work to elicit a response or shared similarities in their structure to either male lures or the reported spiroacetals. Additional compounds from fruit and fermentation that were observed to give robust responses in maxillary palps of tephritid species were also included to anchor the observations in previous work (Larsson‐Herrera et al. 2024). The blends were thus diverse and contained esters, terpenoids, spiroacetals, pyrazines, phenols, and phenylpropanoids (Table S1).

Chemical standards were analyzed prior to being combined into blends and injected at both 100 ng/μl and 10 ng/μl using a GC–MS (Agilent 6890 GC and 5975 MS, Agilent Technologies Inc., Santa Clara, CA, USA), using a polar DB‐WAX column of 60 m × 0.25 mm × 0.25 μm film thickness, with helium as carrier gas. Injection was in splitless mode at 250°C. The oven temperature program was as follows: 50°C for 1.5 min, ramping at 7°C/min to 250°C, hold for 5 min. The lower concentration was used to separate compounds, and the higher concentration was used to parse out responses to synthetics from impurities. Major impurities were tentatively identified, and main compounds were confirmed using the NIST 20 library in masshunter and NIST MS search v. 2.4 as well as published Kovats retention indices. Several synthetic compounds, such as farnesene, were excluded at this step and did not form part of the 40 final compounds due to containing large amounts of impurities. Three blends were constructed, assuring non‐overlapping peaks of the synthetics, and again injected into the GC–MS for verification. An aliphatic alkane solution of C7‐C30 was also injected to calculate Kovats retention indices.

### 2.4 Electrophysiological Experiments

Gas chromatography (GC) (Agilent Technologies 6890 GC (Santa Clara, CA, USA)) coupled with a flame ionization detector (FID) and an electropalpographic detector (EPD) was used to record olfactory responses from the palps of the five tephritid species. For the recording, the insect was immobilized in a 200 μL micropipette tip with the palps exposed. Glass capillary electrodes filled with Beadle‐Ephrussi Ringer's solution (7.5 g NaCl, 0.35 g KCl, 0.29 g CaCl2 dissolved in 1 L of distilled water) were used to record signals from the distal position of the palp against a reference electrode on the head. Three blends were tested at 10 ng/μL, and signals were acquired using GC‐EAD 2014 software (V.1.2.3, Syntech, Kirchzarten, Germany). The GC was equipped with a DB‐WAX column (30 m × 0.25 mm × 0.25 μm, same method described above for the GC–MS), with hydrogen as the carrier gas. The effluent was split equally (1:1) between the FID and the EPD. Males and females of 10–20 days old were subjected to the three blends, with 2 μL injected per trial, and recordings across the blends were to a large extent performed on the same individual. For each blend, three to seven recordings were used, depending on the quality of recording throughout the run. GC‐EPD active peaks were confirmed by comparing Kovats retention indices from the GC‐EPD with GC–MS and published literature.

### 2.5 Analysis

Electrophysiological data were annotated using GC‐EAD software and exported as CSV files. The data were then added to Google Sheets, with one workbook per mix and one worksheet per species. The data were read into R (v. 4.2.2) using the package “googledrive” (D'Agostino McGowan and Bryan 2020). EPD responses were analyzed as relative values to account for inter‐individual and inter‐species differences in electrical properties. Raw mV readings were log‐transformed, and a baseline was computed for each experimental group. Absolute values were scaled to this baseline and further normalized by dividing by the mean scaled response, yielding a final relative metric. Species differences for each compound were tested using one‐way ANOVA, followed by post hoc Tukey's HSD tests where appropriate. Sex differences within each species–compound combination were evaluated using independent‐samples *t*‐tests. All *p*‐values were adjusted for multiple comparisons using the Benjamini–Hochberg method. Compounds were resolved against PubChem using webchem (Szöcs et al. 2020) and quality controlled for annotation discrepancies. Unknown compounds were given the name “unknown” plus their retention index. The biosynthetic pathway for each compound was resolved using SMILES through the API of NPClassifier (Kim et al. 2021), with the exception of spiroacetals, which were manually labeled. Linear models were constructed for each of the three major pathways of esters, shikimates, and phenylpropanoids, and spiroacetals across all pairwise combinations of insect species. Dendrograms of responses were constructed using a Jaccard dissimilarity index from the package “vegan” (Oksanen et al. 2022). A phylogenetic analysis was constructed by concatenating the 16 s and COI gene sequences, using data from Virgilio et al. (2015), with *Acanthiophilus helianthi* as an outgroup; sequences were aligned using MAFFT (Katoh and Standley 2013), and a consensus tree was constructed using “iqtree2” (Minh et al. 2020). All dendrograms were plotted using Yu et al. (2017) “ggtree” (), and all other plots and data manipulation were performed with “tidyverse” package (Wickham et al. 2019).

## 3 Results

**FIGURE 1:**
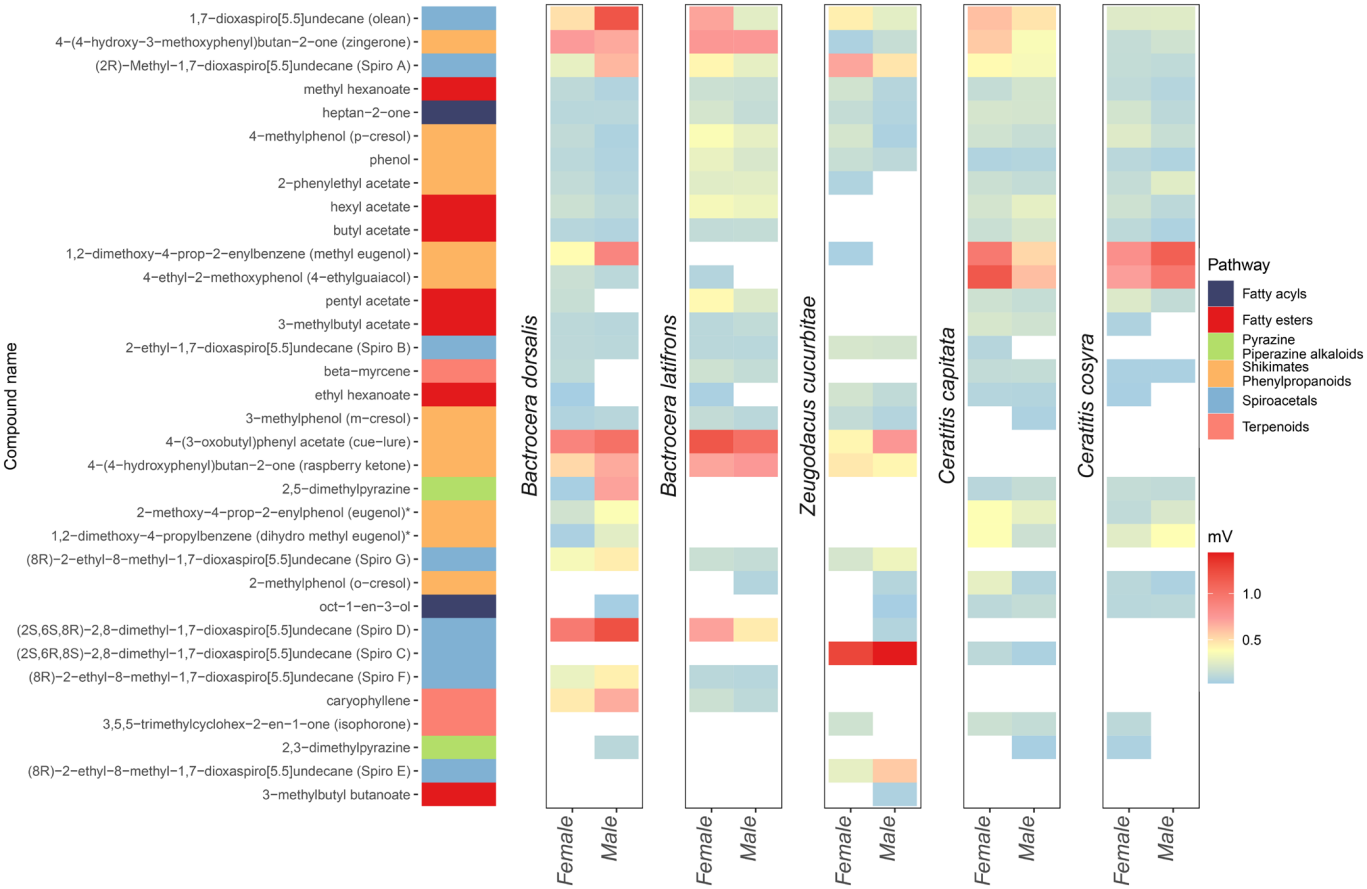
Heatmap of maxillary palp olfactory sensitivities in *Bactrocera dorsalis* , *Bactrocera latifrons* , *Zeugodacus cucurbitae*, *Ceratitis capitata* and *Ceratitis cosyra* to synthetic compounds and two impurities (indicated with an *). From left to right: a) synthetic compounds of the three blends, b) their functional classes, c) olfactory sensitivities of each species to chemical compounds, d) chemical groups used for compound classification, and e) the normalized sensitivity of the fly responses ranging from light blue (0) to red (> 1 mv). The compounds are sorted from top to bottom in decreasing order of sharedness across tephritids species and within each cluster of males and females of a single species.

The maxillary palps of *B. latifrons* , *B. dorsalis* , *Z. cucurbitae*, *C. capitata* , and *C. cosyra* detected 32 out of the 40 synthetic compounds in the synthetic mixes and consistently responded to an additional 36 impurity compounds, of which 34 could not be reliably identified (Figure 1, Figure S1). Compounds were generally detected by both sexes, with a detection overlap ranging from 87.5% in *B. latifrons* to 68% in *Z. cucurbitae*. Responses to compounds detected by only one sex were consistently weaker (< 0.2 mV).

In all species, the three compounds that elicited the strongest responses were either spiroacetals or phenylpropanoids. However, these differed between species: for *B. dorsalis*, the strongest responses were elicited by spiro D, cue‐lure, and olean, whereas in *B. latifrons* by cue‐lure, zingerone, and raspberry ketone, and in *Z. cucurbitae by* spiro C, spiro A, and cue‐lure. Both *Ceratitis* species responded most strongly to methyl eugenol and 4‐ethyl guaiacol, followed by olean and dihydro methyl eugenol, an impurity, for *C. capitata* and *C. cosyra* , respectively.

All species detected the fatty acyl heptan‐2‐one, and all, except *B. latifrons*, detected 1‐octen‐3‐ol. While *Z. cucurbitae* detected three fatty esters, all other species detected six. Pyrazines (2,3 and 2,5‐dimethylpyrazines) were only detected by *B. dorsalis* , *C. capitata* , and *C. cosyra* . *B. dorsalis* and *C. capitata* detected the most compounds from the shikimates and phenylpropanoids pathway, with 11 and 10 compounds, respectively, while all the other species detected 9. Only the two *Bactrocera* species detected caryophyllene, while *Z. cucurbitae* and both *Ceratitis* spp. detected isophorone. With the exception of *Z. cucurbitae*, all species detected beta‐myrcene. In contrast, the following compounds were not detected by the palps of any species: (*E*)‐4,8‐dimethyl‐1,3,7‐nonatriene (DMNT), 2‐phenethyl propionate, 2‐methylpropyl 3‐methylbutanoate, 3‐hydroxy‐2‐butanone (acetoin), limonene, trimedlure, alpha‐terpinyl acetate, and beta‐ocimene.

### 3.1 Sensitivity to Spiroacetals

**FIGURE 2:**
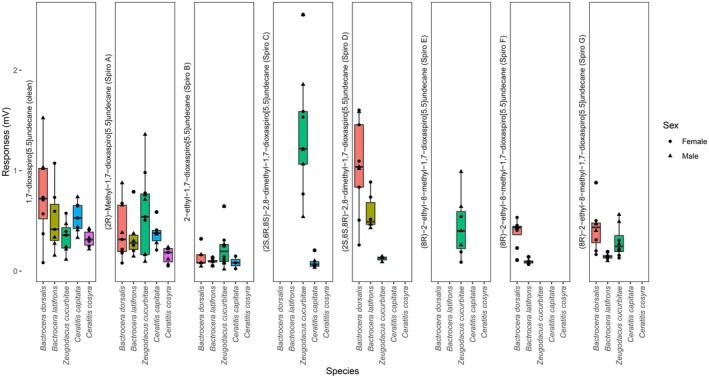
Amplitude of olfactory responses of male and female maxillary palps of five tephritid species ( *Bactrocera dorsalis* , *Bactrocera latifrons* , *Zeugodacus cucurbitae*, *Ceratitis capitata* , and *Ceratitis cosyra* ) toward spiroacetals, including olean, and synthesized spiro A, spiro B, spiro C, spiro D, spiro E, spiro F, spiro G, either emitted or shared between few tephritid species.

The five species showed both overlapping and differential palpal responses toward the eight spiroacetals in the panel (Figure 2). The two simplest forms of spiroacetals, olean and spiro A, were detected by all species and both sexes. *Ceratitis cosyra* detected the least number of spiros, only olean and spiro A. *Zeugodacus cucurbitae*, on the other hand, detected all spiroacetals except spiro F. *B. dorsalis* and *B. latifrons* detected the same six spiroacetals, albeit with a differential response strength. Notably, spiro B elicited weak responses (< 0.25 mV) across all species, except *C. cosyra* , which did not detect this compound. Some spiroacetals were only detected by a few species, such as spiro C, which was detected exclusively by *Z. cucurbitae* and *C. capitata* , evoking strong (> 1 mV) and weak (< 0.25 mV) responses, respectively. In addition, the two *Bactrocera* species responded most strongly to spiro D, followed by olean. Spiro D and spiro G were detected by *Z. cucurbitae*, *B. latifrons* , and *B. dorsalis* . While Spiro F was detected solely by the two *Bactrocera* species, only *Z. cucurbitae* detected spiro E. Spiro E, spiro F, and spiro G are chiral isomers of 2‐ethyl‐8‐methyl‐1,7‐dioxaspiro[5.5]undecane racemate, for which the chirality could not be resolved.

### 3.2 Olfactory Sensitivities to Phenylpropanoids (Male Lures)

**FIGURE 3:**
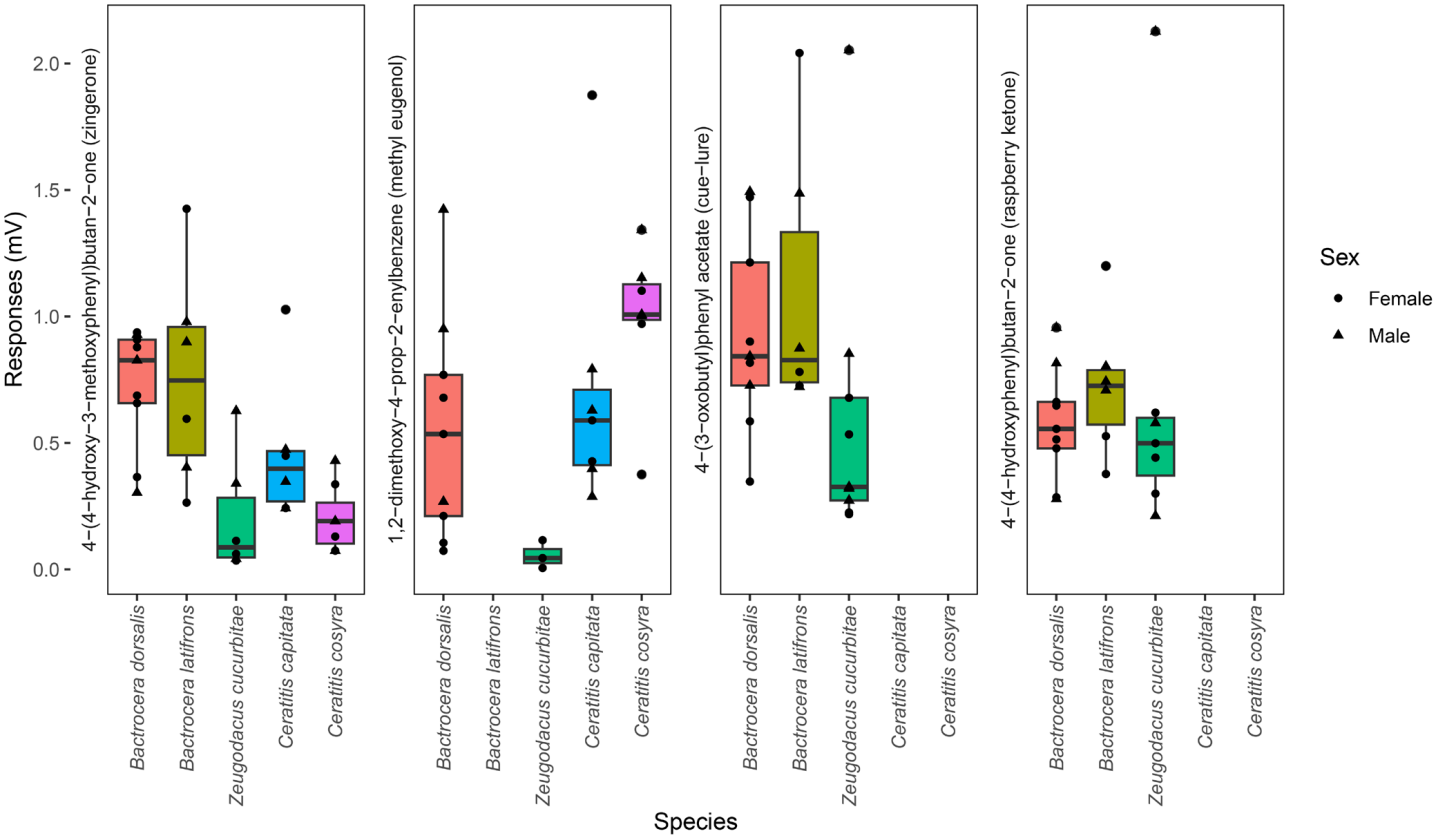
Amplitude of olfactory responses of male and female maxillary palps of five tephritid species ( *Bactrocera dorsalis* , *Bactrocera latifrons* , *Zeugodacus cucurbitae*, *Ceratitis capitata* , and *Ceratitis cosyra* ) toward phenylpropanoids (zingerone, methyl eugenol (ME), cuelure (CL), raspberry ketone (RK)), known as male lures for many *Bactrocera* species.

Significant interspecific differences in EPD responses were detected for some male‐lure compounds (ANOVA: methyl eugenol, *F*(3,22) = 3.75, *p* = 0.026; zingerone, *F*(4,29) = 6.58, *p* = 0.0007), but no sex‐based differences for any species‐compound combination (all *p_adj* ≥ 0.919). For ME, post hoc tests indicated that *Z. cucurbitae* responded significantly less than *C. cosyra* (*p*_adj = 0.019), with non‐significant trends toward lower responses compared to *B. dorsalis* (*p* = 0.305) and *C. capitata* (*p* = 0.880). Consistent with this, the three species with strong ME responses ( *B. dorsalis* , *C. capitata* , and *C. cosyra* ) were also sensitive to similar compounds such as eugenol and the impurity dihydro methyleugenol, whereas *Z. cucurbitae* and *B. latifrons* did not detect these (**Figure** **3**). In contrast, zingerone was detected by all species, though with significant variation in sensitivity. *B. dorsalis* and *B. latifrons* responded more strongly than *Z. cucurbitae* and *C. cosyra* (all *p*_adj < 0.012). No interspecific differences were observed for raspberry ketone (RK) or cuelure (CL) (*p* > 0.17), which were only detected by the two *Bactrocera* species and *Z. cucurbitae*. Trimedlure and terpinyl acetate were not detected by any species. Moreover, only *Z. cucurbitae* and the two species of *Ceratitis* detected isophorone.

### 3.3 Correlation Between Tephritids Palpal Olfactory Sensitivities to Spiroacetals, Phenylpropanoids and Esters

**FIGURE 4:**
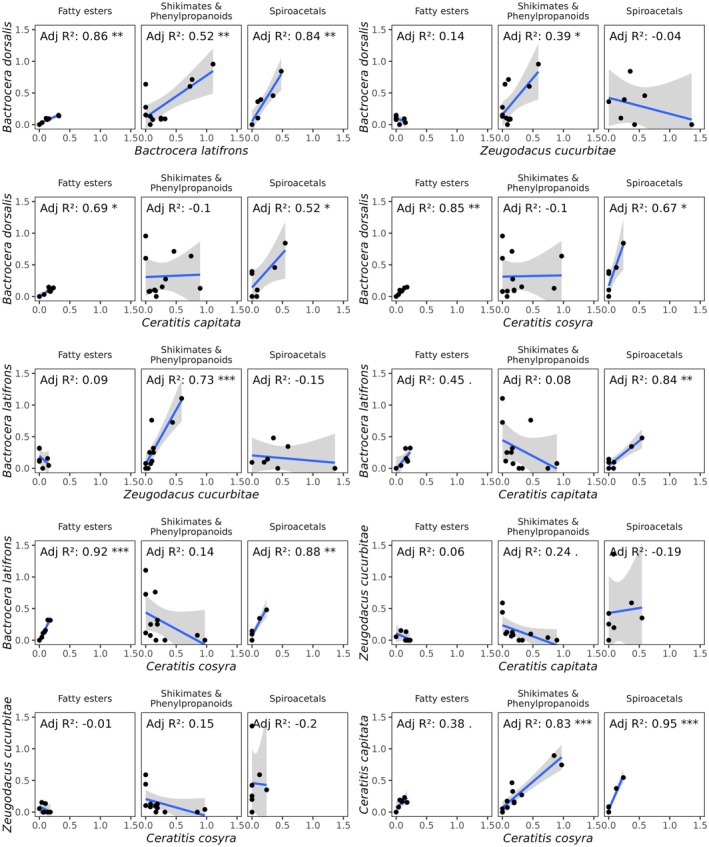
Linear models for olfactory responses of maxillary palps of the five tephritid species ( *Bactrocera dorsalis* , *Bactrocera latifrons* , *Zeugodacus cucurbitae*, *Ceratitis capitata* , and *Ceratitis cosyra* ) to esters, shikimates, and phenylpropanoids and spiroacetals, as well as across all pairwise combinations. Stars highlight the level of significance * < 0.05, ** < 0.01, *** < 0.001.

Comparison between species showed that receptive ranges and strength of palpal responses were significantly correlated between *B. dorsalis* and *B. latifrons* for esters (*p* < 0.01, *R* ^2^ = 0.86), phenylpropanoids (*p* < 0.01, *R* ^2^ = 0.52), and spiroacetals (*p* < 0.01, *R* ^2^ = 0.84, Figure 4). In a similar way, *C. capitata* and *C. cosyra* were also significantly correlated for phenylpropanoids (*p* < 0.001, *R* ^2^ = 0.83) and spiroacetals (*p* < 0.001, *R* ^2^ = 0.95). While many correlations were found between *B. latifrons* / *B. dorsalis* and *C. cosyra/C. capitata* for esters and spiroacetals, no correlation was observed between *Z. cucurbitae* and *C. cosyra* / *C. capitata* .

**FIGURE 5:**
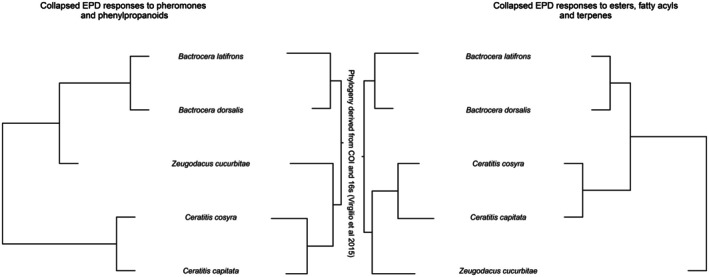
On the left: A tree based on EPD responses of five tephritid species ( *Bactrocera dorsalis* , *Bactrocera latifrons* , *Zeugodacus cucurbitae*, *Ceratitis capitata* , and *Ceratitis cosyra* ) to spiroacetals and phenylpropanoids. Since the responses did not consistently differ between sexes, the data were combined. On the right: A tree based on collapsed EPD responses to general odors (esters, fatty acyls, and terpenes). In the center: A phylogenetic tree of the five tephritids plotted using phylogenetic data concatenated from COI and 16 s, based on a subset of data from Virgilio et al. (2015). Whereas responses to compounds from the spiroacetal and phenylpropanoid pathways followed phylogeny, responses to compounds from fatty acid and terpenoid pathways formed a cluster that correlates with ecological niche.

Finally, a consensus tree of EPD responses of the five species to pheromones (spiroacetals) and parapheromones (male lures) using a dissimilarity index (Jaccard) closely aligned with a consensus phylogenetic tree derived from mitochondrial (COI) and ribosomal (16S) DNA, whereas EPD responses of the five species to general odors (which were primarily fatty acid and terpenoid derivatives) clustered separately, and this grouping correlated with their ecological niches (Figure 5).

## 4 Discussion

The maxillary palps of Tephritidae are increasingly studied, from OSN morphology (Zhang et al. 2011; Chieng et al. 2018; Liu et al. 2020; Larsson‐Herrera et al. 2024) to functional studies using EPGs and EPDs (Chieng et al. 2018; Verschut et al. 2018; Oh et al. 2019; Biswas et al. 2020; Noushini, Park, et al. 2020; Noushini, Perez, et al. 2020; Noushini et al. 2021; Larsson‐Herrera et al. 2024, this study). This growing attention is particularly due to the finding that the palps of tephritids mediate detection and attraction to pheromones and parapheromones (Giannakakis and Fletcher 1981; Metcalf et al. 1983), which are important in monitoring and control (Tan et al. 2014; Scolari et al. 2021). Yet, the receptive range of maxillary palps of Tephritidae is broader than just (para)pheromones and includes robust responses to fruit and food volatiles (Larsson‐Herrera et al. 2024).

Odor detection by the maxillary palps of tephritid flies, with a sensitivity to general odors and odors that induce sexually divergent behaviors, appears to serve multiple ecological functions that likely involve segregated neural processing pathways. While both odor classes activate palpal OSNs, (para)pheromones may engage conserved lateral horn circuits driving hardwired attraction behaviors, whereas food odors could integrate into mushroom body networks for context‐dependent valuation (Dweck et al. 2016; Giurfa 2013). These functional differences may subject palpal detection systems to divergent selection pressures. For food odors, directional selection may optimize sensitivity to dominant host volatiles (e.g., fruit terpenes), while stabilizing selection may preserve high‐affinity receptors for trace (para)pheromones due to their reproductive significance (Gonzalez et al. 2020). Temporal niche partitioning (e.g., crepuscular pheromone release vs. diurnal feeding) likely minimizes masking effects between these odor classes.

The current study shows that, while the palps indeed respond to fruit and food odors, they are particularly sensitive to (para)pheromones, with a surprising breadth and cross sensitivity across taxa that do not reflect behavioral sensitivity. In addition, a cluster analysis showed that the responses to parapheromones closely followed phylogeny rather than ecology, and thereby differed from fruit and food odor sensitivities, which followed ecology rather than phylogeny (Biasazin et al. 2019; Larsson‐Herrera et al. 2024, this study). Below, the results are discussed in the context of (para)pheromone production, known behavioral responses, and the evolutionary ecology of Tephritidae.

### 4.1 Sensitivity to Male Lures or Parapheromones

*Bactrocera* and *Zeugodacus* spp. are generally classified into three categories based on their behavioral responses to phenylpropanoids: ME responsive, RK/CL responsive, and non‐lure responsive (Clarke 2019; Royer et al. 2017; Drew et al. 1982; Metcalf and Metcalf 1992; Tan et al. 2010). How the olfactory circuitry regulates these, often very strong, male‐specific behavioral responses is not understood, and neither is the evolutionary ecology of the sensitivity to these compounds.

Whereas ablation experiments show the importance of maxillary palps, the receptive ranges of the maxillary palps to these compounds across species are generally not known. Our data provide some important correlates that warrant further study. As the palpal minicircuitry consists of only six OSN types, several of which mediate responses to a wide range of chemically diverse fruit and food odors (including esters, terpenes, ketones, pyrazines, as well as spiroacetals, Larsson‐Herrera et al. 2024, this study), the number of OSNs types mediating phenylpropanoid responses would have to be very limited. Possibly, only one OSN type detects phenylpropanoids and responses may thus reflect the tuning breadth of a single OSN that is critical in mediating male attraction. In the maxillary palps of *D. melanogaster* , a single OSN class (pb1b) also displays a strong sensitivity to phenylpropanoids, with two other neurons being sensitive to other phenolics (de Bruyne et al. 1999; Dweck et al. 2016). However, no particular role of phenylpropanoids or other phenolics has been described in the ecology of drosophilids.

Surprisingly, however, palpal sensitivity to phenylpropanoids was not always congruent with behavioral sensitivity. For instance, whereas the palps of *B. dorsalis* are essential for inducing the male behavioral response to ME (Chieng et al. 2018), they were also broadly sensitive to other phenylpropanoids that are not male lures for this species. Conversely, the palps of *B. latifrons* , a “non‐lure responsive” species (Clarke 2019), while not sensitive to ME, responded strongly to RK and CL, even stronger than in *Z. cucurbitae*, a species routinely monitored using RK and CL (Kawashita et al. 2004). Similarly, despite not being behaviorally responsive to ME, both *Ceratitis* species exhibited strong responses to this compound, while displaying no palpal sensitivity to their own male lures, trimedlure or α‐terpinyl acetate (Ripley and Hepburn 1935; Khan et al. 2021). Finally, whereas zingerone, RK and CL are known attractants for *Zeugodacus* species (Inskeep et al. 2018; Tan and Nishida 2024), the maxillary palps of *Z. cucurbitae* were relatively insensitive to these phenylpropanoids compared to both *Bactrocera* species, for which these compounds are likely behaviorally insignificant.

These apparent mismatches should be interpreted with caution. Our electrophysiological assays tested compounds in isolation, whereas natural (para)pheromonal signals typically occur as multi‐component blends in precise ratios that determine behavioral valence. Peripheral neuronal firing to an individual compound, therefore, does not necessarily predict attraction, as central processing in higher olfactory centers ultimately determines behavioral output.

Thus, whereas the maxillary palps are important in mediating the behavioral response to male lures (Chieng et al. 2018; Verschut et al. 2018), palpal tuning did not match behavioral sensitivity to (para)pheromones. As palpal sensitivity overlaps with antennal sensitivity, it would seem logical that, to elicit the full sequence of behaviors that lead to for example, trap catches, input from both organs is required (Biasazin et al. 2025), and that this combination of input also renders behavioral specificity. Indeed, the combination of the maxillary palps and the antennae appears critical for the strong behavioral responses to male lures of *B. dorsalis* and *Z. cucurbitae* (Chieng et al. 2018; Verschut et al. 2018). Along the same lines, the palps of *B. latifrons* did not detect isophorone, a reported male attractant that is not nearly as attractive for *B. latifrons* as ME for *B. dorsalis* (Ishida et al. 2008). Similarly, trimedlure and alpha‐terpinyl acetate were not detected by the palps and are not as effective lures for *Ceratitis* species as ME for *B. dorsalis* (Vargas et al. 2012). To fully understand how palpal responses to various compounds lead to behaviors, a solid understanding of the antennal circuitry would seem important.

### 4.2 Spiroacetals

Although species differ in the spiroacetals they produce, the palpal sensitivities overlapped seemingly independent of that. For instance, the simplest spiroacetal tested here, olean (racemic 1,7‐dioxaspiro[5.5]undecane, the major pheromone of the olive fruit fly), was detected by all species in this study, even though olean is not reported from any of these species (Baker et al. 1980). Similarly, spiro A and spiro B were also detected by all or almost all species, respectively, while spiroacetals have not been reported from Tephritidae (Booth et al. 2009). Spiro C, a widespread spiroacetal that is commonly found in *Bactrocera* and *Zeugodacus* species (Baker and Bacon 1985; Zhang et al. 1997; Francke and Kitching 2001), was detected only by *Z. cucurbitae* and slightly by *C. capitata* , while its stereoisomer and uncommon spiro D (Francke and Kitching 2001) was detected by *B. dorsalis* and *B. latifrons* . Spiro E, F, and G, a racemic mixture of 2‐ethyl‐8‐methyl‐1,7‐dioxaspiro[5.5]undecane and identified from several *Bactrocera species* (Booth et al. 2009; El‐Sayed et al. 2019; Noushini, Park, et al. 2020; Noushini, Perez, et al. 2020; Noushini et al. 2021), induced a mosaic sensitivity pattern across the five species, indicating a differential effect of chirality on receptor sensitivity. In this context, it is important to note that spiroacetals are well‐known pheromones not only of Tephritidae but also widely reported from across insect orders (Booth et al. 2009). This may indicate a deeply rooted, ancient OSN and OR system for spiroacetals in insects and, given the abundance and overlap in production across insect lineages, a lower behavioral specificity than what perhaps the connotation of the word “pheromone” might suggest. Further research should look at the OSNs, their ORs underlying spiroacetal responses, as well as the cross‐sensitivity of the OSNs and ORs to various spiroacetals and phenolics. In addition, given the sensitivity of *Ceratitis* species, the potential production of spiroacetals in this and related genera would seem warranted.

### 4.3 EPD Responses to Pheromones and Phenylpropanoids Align With Phylogeny

Previous studies demonstrated that ecology is of overriding importance in the overall olfactome tuning of the antennae (Biasazin et al. 2019) and the maxillary palps (Larsson‐Herrera et al. 2024) to general fruit and food odors. This study provides evidence that, in spite of phylogenetic distance, the sensory responses to these general odors were highly similar between species with a similar ecological niche, even though phylogenetically distant. However, a diametrically opposite pattern emerged for responses to pheromones and parapheromones, which instead closely matched phylogenetic relatedness.

Apparently, opposing selection pressures shape the olfactory circuitry to either niche odors, resulting in directional selection that shows a pattern correlating with ecology, or odors that mediate (para)pheromone communication, resulting in some form of stabilizing selection which consequently follows phylogeny. This contrasting pattern spans tens of millions of years of olfactory evolution of Tephritidae (Zhang et al. 2023) and emerges by virtue of GC‐coupled olfactome measurements, which provide the sums of olfactory sensitivity for a large number of odorants. Indeed, such correlations have not previously been reported from other functional studies on OSNs or ORs. As single sensillum studies generally focus on single or small subsets of OSNs (de Bruyne et al. 2009; Stensmyr et al. 2003; Prelic et al. 2022), overall sensitivities cannot easily be extracted. Similarly, receptor studies that describe the tuning curves of individual ORs are difficult to translate into ensemble in vivo responses (Reisert and Restrepo 2009) and are difficult to understand in terms of selection regimes to which they are subjected.

Besides surfacing evo‐eco patterns of ORs, these evolutionary correlates can be used to direct OR studies. Given the limited number of ORs expressed in the palps, one could use the opposing selection pressures on (sets of) ORs to tease out which palpal ORs putatively respond to general odors versus those that respond to (para)pheromones. Based on the differential selection pressures, one would expect sequences of (para)pheromone‐sensitive ORs to more strictly follow phylogeny than ORs sensitive to general odors. Additionally, this may provide insights into the amino acid sequence(s) that are involved in ligand binding and give rise to these differential responses, particularly for ORs under directional selection amidst an abundance of sequence information following phylogeny (Saad et al. 2018).

## 5 Conclusions and Further Research

The functional characterization of the maxillary palps of Tephritidae provides important insights into the circuitry underlying (para)pheromones, and how detection may, or may not, steer behavior toward these compounds. It appears that the detection of (para)pheromones by the maxillary palps is not an indication of behavioral sensitivity. While this supports the idea that behavioral responses rely on a synergy between palpal and antennal input in mediating the full range of behaviors, it also raises the question of how such synergy is mediated and which OSN/OR combinations are responsible for this integration. Further questions of interest include, for instance, whether such (sub)circuits exist in non‐responsive species or non‐responsive sexes, and whether they can be activated through hitherto undescribed odor combinations that induce responses in OSN types that are part of this circuitry. The data also provide novel insights of how differential selection pressures shape the evolution of olfactory sensitivities and follow either ecology or phylogeny. The emerging patterns may be useful in further unraveling which ORs underlie responses to which odors, as well as guide future studies deciphering ORs' function by indicating putative sequences that may be involved in ligand binding. Finally, knowing the sensitivities of the palps could be used in designing novel odor combinations for testing in field trials.

## Author Contributions

**Chaymae Fennine:** conceptualization (equal), data curation (equal), formal analysis (equal), methodology (equal), visualization (equal), writing – original draft (equal), writing – review and editing (equal). **Sebastian Larsson Herrera:** conceptualization (equal), data curation (equal), formal analysis (equal), methodology (equal), visualization (equal), writing – original draft (equal), writing – review and editing (equal). **Tibebe Dejene Biasazin:** conceptualization (equal), funding acquisition (equal), investigation (equal), methodology (equal), validation (equal), writing – review and editing (equal). **Wittko Francke:** conceptualization (equal), investigation (equal), methodology (equal). **Sergio Angeli:** conceptualization (equal), supervision (equal), writing – review and editing (equal). **Teun Dekker:** conceptualization (equal), data curation (equal), formal analysis (equal), funding acquisition (equal), methodology (equal), project administration (equal), resources (equal), supervision (equal), validation (equal), visualization (equal), writing – original draft (equal), writing – review and editing (equal).

## Conflicts of Interest

The authors declare no conflicts of interest.

## Supporting information


**Table S1:** Chemical compounds of the three blends (10 ng) of VOCs (40 compounds) used for electrophysiological recordings on maxillary palps of males and females of *Bactrocera dorsalis* , *Bactrocera latifrons* , *Zeugodacus cucurbitae*, *Ceratitis capitata* and *Ceratitis cosyra* . The blends were constructed based on the Kovats indices of the synthetic compounds to avoid overlap during elution.

## Data Availability

All data utilized in this manuscript, along with the scripts to generate the figures, are available for review at https://zenodo.org/records/14893742?preview=1&token=eyJhbGciOiJIUzUxMiIsImlhdCI6MTczOTk4MDAyOSwiZXhwIjoxNzY3MTM5MTk5fQ.eyJpZCI6IjM2NGQzNjQyLTVhNTItNGY3Mi1hNmI1LWE4MGY0ZmRkM2VhMSIsImRhdGEiOnt9LCJyYW5kb20iOiJkOWFlZjg3N2VhZDIyNTE0MWNlOGJhYzVhMDllZDY1YSJ9.oAILIUqJGFNsLJm4B1BlOIyX3q7Nzzq_fLJdeLzARHMg0jnODB45Qdu3_muD25BWzeliHQN2EHygmTaf8WW6eA.

## References

[ece372261-bib-0001] Baker, R. , and A. J. Bacon . 1985. “The Identification of Spiroacetals in the Volatile Secretions of Two Species of Fruit Fly ( *Dacus dorsalis* , *Dacus Curcurbitae*).” Experientia 41: 1484–1485. 10.1007/bf01950049.

[ece372261-bib-0002] Baker, R. , R. Herbert , P. E. Howse , O. T. Jones , W. Francke , and W. Reith . 1980. “Identification and Synthesis of the Major Sex Pheromone of the Olive Fly (*Dacus Oleae*).” Journal of the Chemical Society, Chemical Communications 2: 52–53. 10.1039/c39800000052.

[ece372261-bib-0003] Beroza, M. , N. Green , S. I. Gertler , L. F. Steiner , and D. H. Miyashita . 1961. “Insect Attractants, New Attractants for the Mediterranean Fruit Fly.” Journal of Agricultural and Food Chemistry 9: 361–365. 10.1021/jf60117a007.

[ece372261-bib-0004] Biasazin, T. D. , H. T. Chernet , S. L. Herrera , et al. 2018. “Detection of Volatile Constituents From Food Lures by Tephritid Fruit Flies.” Insects 9: 119. 10.3390/insects9030119. 30223498 PMC6163689

[ece372261-bib-0005] Biasazin, T. D. , S. Larsson Herrera , F. Kimbokota , and T. Dekker . 2019. “Translating Olfactomes Into Attractants: Shared Volatiles Provide Attractive Bridges for Polyphagy in Fruit Flies.” Ecology Letters 22: 108–118. 10.1111/ele.13172. 30370646

[ece372261-bib-0006] Biasazin, T. D. , R. N. Miano , X. Cheseto , S. Ndlela , S. A. Mohamed , and T. Dekker . 2025. “Selective Lure for *Bactrocera Dorsalis* Based on Shared Palpal and Antennal Responses Across Three Economically Important Tephritid Species.” Journal of Pest Science: 1–13. 10.1007/s10340-025-01898-y.

[ece372261-bib-0007] Biasazin, T. D. , T. W. Wondimu , S. L. Herrera , et al. 2021. “Dispersal and Competitive Release Affect the Management of Native and Invasive Tephritid Fruit Flies in Large and Smallholder Farms in Ethiopia.” Scientific Reports 11: 2690. 10.1038/s41598-020-80151-1. 33514782 PMC7846734

[ece372261-bib-0008] Biswas, M. J. H. , B. Mainali , S. J. Park , P. Taylor , and P. Rempoulakis . 2020. “Electrophysiological Responses to Cuelure of Raspberry Ketone‐Fed Queensland Fruit Flies.” Journal of Economic Entomology 113: 2832–2839. 10.1093/jee/toaa242. 33111947

[ece372261-bib-0009] Booth, Y. K. , W. Kitching , and J. J. De Voss . 2009. “Biosynthesis of Insect Spiroacetals.” Natural Product Reports 26: 490–525. 10.1039/b717392j. 19642419

[ece372261-bib-0010] Booth, Y. K. , B. D. Schwartz , M. T. Fletcher , L. K. Lambert , W. Kitching , and J. J. De Voss . 2007. “A Diverse Suite of Spiroacetals, Including a Novel Branched Representative, Is Released by Female *Bactrocera Tryoni* (Queensland Fruit Fly).” ChemInform 38: chin.200707187. 10.1002/chin.200707187. 17003870

[ece372261-bib-0011] Chieng, A. C.‐T. , A. K.‐W. Hee , and S.‐L. Wee . 2018. “Involvement of the Antennal and Maxillary Palp Structures in Detection and Response to Methyl Eugenol by Male *Bactrocera Dorsalis* (Diptera: Tephritidae).” Journal of Insect Science 18: 19. 10.1093/jisesa/iey104. PMC6197378 30351432

[ece372261-bib-0012] Clarke, A. R. 2019. Biology and Management of Bactrocera and Related Fruit Flies. CABI.

[ece372261-bib-0013] D'Agostino McGowan, L. , and J. Bryan . 2020. “Googledrive: An Interface to Google Drive, R Package Version 1.0.1.” https://googledrive.tidyverse.org/.

[ece372261-bib-0014] de Bruyne, M. , P. J. Clyne , and J. R. Carlson . 1999. “Odor Coding in a Model Olfactory Organ: The *Drosophila* Maxillary Palp.” Journal of Neuroscience 19: 4520–4532. 10.1523/jneurosci.19-11-04520.1999. 10341252 PMC6782632

[ece372261-bib-0015] de Bruyne, M. , R. Smart , E. Zammit , and C. G. Warr . 2009. “Functional and Molecular Evolution of Olfactory Neurons and Receptors for Aliphatic Esters Across the *Drosophila* Genus.” Journal of Comparative Physiology A 196: 97–109. 10.1007/s00359-009-0496-6. 20033746

[ece372261-bib-0016] Drew, R. A. I. , G. H. S. Hooper , and M. A. Bateman . 1982. Economic Fruit Flies of the South Pacific Region. Department of Primary Industries. Qid 4068 and Department of Health., Canberra.

[ece372261-bib-0017] Dweck, H. K. , S. A. Ebrahim , M. A. Khallaf , et al. 2016. “Olfactory Channels Associated With the *Drosophila* Maxillary Palp Mediate Short‐ and Long‐Range Attraction.” ELife 5: e14925. 10.7554/elife.14925. 27213519 PMC4927298

[ece372261-bib-0018] El‐Sayed, A. M. , U. Venkatesham , C. R. Unelius , et al. 2019. “Chemical Composition of the Rectal Gland and Volatiles Released by Female Queensland Fruit Fly, *Bactrocera Tryoni* (Diptera: Tephritidae).” Environmental Entomology 48: 807–814. 10.1093/ee/nvz061. 31145449

[ece372261-bib-0019] Francke, W. , and W. Kitching . 2001. “Spiroacetals in Insects.” Current Organic Chemistry 5: 233–251. 10.2174/1385272013375652.

[ece372261-bib-0020] Giannakakis, A. , and B. S. Fletcher . 1981. “Ablation Studies Related to the Location of the Sex Pheromone Receptors of the Queensland Fruit Fly, *Dacus Tryoni* (Froggat) (Diptera: Tephritidae).” Australian Journal of Entomology 20: 9–12. 10.1111/j.1440-6055.1981.tb00992.x.

[ece372261-bib-0021] Giurfa, M. 2013. “Cognition With Few Neurons: Higher‐Order Learning in Insects.” Trends in Neurosciences 36, no. 5: 285–294. 10.1016/j.tins.2012.12.011. 23375772

[ece372261-bib-0022] Gonzalez, F. , F. Borrero‐Echeverry , J. K. Jósvai , et al. 2020. “Odorant Receptor Phylogeny Confirms Conserved Channels for Sex Pheromone and Host Plant Signals in Tortricid Moths.” Ecology and Evolution 10: 7334–7348. 10.1002/ece3.6458. 32760532 PMC7391548

[ece372261-bib-0023] Haniotakis, G. E. 1974. “Sexual Attraction in the Olive Fruit Fly, *Dacus Oleae* (Gmelin)1.” Environmental Entomology 3: 82–86. 10.1093/ee/3.1.82.

[ece372261-bib-0024] Inskeep, J. R. , H. Spafford , and T. E. Shelly . 2018. “Trapping Male Melon Flies, *Zeugodacus Cucurbitae* (Coquillett) (Diptera: Tephritidae), Using Mixtures of Zingerone and Cue‐Lure in the Field.” Proceedings of the Hawaiian Entomological Society 50: 67–75. http://hdl.handle.net/10125/61791.

[ece372261-bib-0025] Ishida, T. , H. Enomoto , and R. Nishida . 2008. “New Attractants for Males of the Solanaceous Fruit Fly *Bactrocera Latifrons* .” Journal of Chemical Ecology 34: 1532–1535. 10.1007/s10886-008-9562-8. 19018595

[ece372261-bib-0026] Katoh, K. , and D. M. Standley . 2013. “MAFFT Multiple Sequence Alignment Software Version 7: Improvements in Performance and Usability.” Molecular Biology and Evolution 30: 772–780. 10.1093/molbev/mst010. 23329690 PMC3603318

[ece372261-bib-0027] Kawano, Y. , W. C. Mitchell , and H. Matsumoto . 1968. “Identification of the Male Oriental Fruit Fly Attractant in the Golden Shower blossom1.” Journal of Economic Entomology 61: 986–988. 10.1093/jee/61.4.986.

[ece372261-bib-0028] Kawashita, T. , G. B. J. P. Rajapakse , and K. Tsuruta . 2004. “Population Surveys of *Bactrocera* Fruit Flies by Lure Trap in Sri Lanka.” Research Bulletin of the Plant Protection Service, Japan 40: 83–87.

[ece372261-bib-0029] Kent, K. S. , I. D. Harrow , P. Quartararo , and J. G. Hildebrand . 1986. “An Accessory Olfactory Pathway in Lepidoptera: The Labial Pit Organ and Its Central Projections in Manduca Sexta and Certain Other Sphinx Moths and Silk Moths.” Cell and Tissue Research 245, no. 2: 237–245. 10.1007/BF00213927. 3742559

[ece372261-bib-0030] Khan, M. , A. Bari , and M. Hossain . 2021. “Evaluation of Solid Lure Plugs and Insecticide Dispensers on Capturing Dacine Fruit Flies and Non‐Target Insects.” Entomology and Applied Science Letters 8: 35–44. 10.51847/1cxmpdpzjg.

[ece372261-bib-0031] Kim, H. W. , M. Wang , C. A. Leber , et al. 2021. “NPClassifier: A Deep Neural Network‐Based Structural Classification Tool for Natural Products.” Journal of Natural Products 84: 2795–2807. 10.1021/acs.jnatprod.1c00399. 34662515 PMC8631337

[ece372261-bib-0032] Klinner, C. F. , C. König , C. Missbach , et al. 2016. “Functional Olfactory Sensory Neurons Housed in Olfactory Sensilla on the Ovipositor of the Hawkmoth *Manduca sexta* .” Frontiers in Ecology and Evolution 4: 130. 10.3389/fevo.2016.00130.

[ece372261-bib-0033] Larsson‐Herrera, S. , F. Kimbokota , S. Ahmad , K. Heise , T. D. Biasazin , and T. Dekker . 2024. “The Maxillary Palps of Tephritidae Are Selectively Tuned to Food Volatiles and Diverge With Ecology.” Journal of Insect Physiology 154: 104632. 10.1016/j.jinsphys.2024.104632. 38531436

[ece372261-bib-0034] Liu, Y. , J. He , R. Zhang , and L. Chen . 2020. “Sensilla on Antenna and Maxillary Palp of *Neoceratitis asiatica* (Diptera: Tephritidae).” Micron 138: 102921. 10.1016/j.micron.2020.102921. 32818763

[ece372261-bib-0035] Metcalf, R. L. , and E. R. Metcalf . 1992. Plant Kairomones in Insect Ecology and Control. Springer.

[ece372261-bib-0036] Metcalf, R. L. , W. C. Mitchell , and E. R. Metcalf . 1983. “Olfactory Receptors in the Melon Fly *Dacus Cucurbitae* and the Oriental Fruit Fly *Dacus dorsalis* .” Proceedings of the National Academy of Sciences 80: 3143–3147. 10.1073/pnas.80.11.3143. PMC393996 16593321

[ece372261-bib-0037] Minh, B. Q. , H. A. Schmidt , O. Chernomor , et al. 2020. “IQ‐TREE 2: New Models and Efficient Methods for Phylogenetic Inference in the Genomic Era.” Molecular Biology and Evolution 37: 1530–1534. 10.1093/molbev/msaa015. 32011700 PMC7182206

[ece372261-bib-0038] Missbach, C. , H. K. Dweck , H. Vogel , et al. 2014. “Author Response: Evolution of Insect Olfactory Receptors.” eLife 3: e02115. 10.7554/elife.02115.030. 24670956 PMC3966513

[ece372261-bib-0039] Noushini, S. , S. J. Park , I. Jamie , J. Jamie , and P. Taylor . 2020. “Rectal Gland Exudates and Emissions of *Bactrocera bryoniae* : Chemical Identification, Electrophysiological and Pheromonal Functions.” Chemoecology 31: 137–148. 10.1007/s00049-020-00335-z.

[ece372261-bib-0040] Noushini, S. , S. J. Park , J. Perez , et al. 2021. “Electrophysiological Responses of *Bactrocera Kraussi* (Hardy) (Tephritidae) to Rectal Gland Secretions and Headspace Volatiles Emitted by Conspecific Males and Females.” Molecules 26: 5024. 10.3390/molecules26165024. 34443611 PMC8399695

[ece372261-bib-0041] Noushini, S. , J. Perez , S. J. Park , et al. 2020. “Attraction and Electrophysiological Response to Identified Rectal Gland Volatiles in *Bactrocera Frauenfeldi* (Schiner).” Molecules 25: 1275. 10.3390/molecules25061275. 32168881 PMC7143976

[ece372261-bib-0042] Oh, H. , S. A. Jeong , J. Kim , and K. C. Park . 2019. “Morphological and Functional Heterogeneity in Olfactory Perception Between Antennae and Maxillary Palps in the Pumpkin Fruit Fly, *Bactrocera Depressa* .” Archives of Insect Biochemistry and Physiology 101: e21560. 10.1002/arch.21560. 31152462

[ece372261-bib-0043] Oksanen, J. , G. L. Simpson , F. G. Blanchet , et al. 2022. Vegan: Community Ecology Package. CRAN: Contributed Packages. 10.32614/cran.package.vegan.

[ece372261-bib-0044] Ono, H. , A. K.‐W. Hee , and H. Jiang . 2021. “Recent Advancements in Studies on Chemosensory Mechanisms Underlying Detection of Semiochemicals in Dacini Fruit Flies of Economic Importance (Diptera: Tephritidae).” Insects 12: 106. 10.3390/insects12020106. 33530622 PMC7911962

[ece372261-bib-0045] Park, K. C. , S. A. Jeong , G. Kwon , and H. Oh . 2018. “Olfactory Attraction Mediated by the Maxillary Palps in the Striped Fruit Fly, *Bactrocera Scutellata*: Electrophysiological and Behavioral Study.” Archives of Insect Biochemistry and Physiology 99: e21510. 10.1002/arch.21510. 30350371

[ece372261-bib-0046] Perkins, M. V. , M. T. Fletcher , W. Kitching , R. A. I. Drew , and C. J. Moore . 1990. “Chemical Studies of Rectal Gland Secretions of Some Species of *Bactrocera Dorsalis* Complex of Fruit Flies (Diptera: Tephritidae).” Journal of Chemical Ecology 16: 2475–2487. 10.1007/bf01017470. 24264212

[ece372261-bib-0047] Prelic, S. , V. Pal Mahadevan , V. Venkateswaran , S. Lavista‐Llanos , B. S. Hansson , and D. Wicher . 2022. “Functional Interaction Between *Drosophila* Olfactory Sensory Neurons and Their Support Cells.” Frontiers in Cellular Neuroscience 15: 789086. 10.3389/fncel.2021.789086. 35069116 PMC8777253

[ece372261-bib-0048] Reisert, J. , and D. Restrepo . 2009. “Molecular Tuning of Odorant Receptors and Its Implication for Odor Signal Processing.” Chemical Senses 34, no. 7: 535–545. 19525317 10.1093/chemse/bjp028 PMC2733323

[ece372261-bib-0049] Ripley, L. B. , and G. A. Hepburn . 1935. Olfactory Attractants for Male Fruit‐Flies. CABI.

[ece372261-bib-0050] Royer, J. E. , S. Agovaua , J. Bokosou , et al. 2017. “Responses of Fruit Flies (Diptera: Tephritidae) to New Attractants in Papua New Guinea.” Austral Entomology 57: 40–49. 10.1111/aen.12269.

[ece372261-bib-0051] Saad, R. , A. B. Cohanim , M. Kosloff , and E. Privman . 2018. “Neofunctionalization in Ligand Binding Sites of Ant Olfactory Receptors.” Genome Biology and Evolution 10, no. 9: 2490–2500. 29982411 10.1093/gbe/evy131 PMC6161762

[ece372261-bib-0052] Schorkopf, D. L. P. , B. P. Molnár , M. Solum , et al. 2019. “False Positives From Impurities Result in Incorrect Functional Characterization of Receptors in Chemosensory Studies.” Progress in Neurobiology 181: 101661. 10.1016/j.pneurobio.2019.101661. 31310789

[ece372261-bib-0053] Scolari, F. , F. Valerio , G. Benelli , N. T. Papadopoulos , and L. Vaníčková . 2021. “Tephritid Fruit Fly Semiochemicals: Current Knowledge and Future Perspectives.” Insects 12: 408. 10.3390/insects12050408. 33946603 PMC8147262

[ece372261-bib-0054] Segura, D. F. , S. A. Belliard , M. T. Vera , et al. 2018. “Plant Chemicals and the Sexual Behavior of Male Tephritid Fruit Flies.” Annals of the Entomological Society of America 111, no. 5: 239–264.

[ece372261-bib-0055] Stensmyr, M. C. , E. Giordano , A. Balloi , A. M. Angioy , and B. S. Hansson . 2003. “Novel Natural Ligands for *Drosophila* Olfactory Receptor Neurones.” Journal of Experimental Biology 206, no. 4: 715–724. 10.1242/jeb.00143. 12517989

[ece372261-bib-0056] Stork, N. E. 2018. “How Many Species of Insects and Other Terrestrial Arthropods Are There on Earth?” Annual Review of Entomology 63, no. 2018: 31–45. 10.1146/annurev-ento-020117-043348. 28938083

[ece372261-bib-0057] Szöcs, E. , T. Stirling , E. R. Scott , A. Scharmüller , and R. B. Schäfer . 2020. “Webchem: An R Package to Retrieve Chemical Information From the Web.” Journal of Statistical Software 93: 1–17. 10.18637/jss.v093.i13.

[ece372261-bib-0058] Tan, K. H. , and R. Nishida . 2024. “A Review on Natural Phenylbutanoid Attractants: Occurrence, Distribution, and Role in Nature, Especially in Relation to Dacini Fruit Fly Behavior and Pollination.” Journal of Chemical Ecology 50: 926–946. 10.1007/s10886-024-01499-6. 38644437

[ece372261-bib-0059] Tan, K. H. , R. Nishida , E. B. Jang , and T. E. Shelly . 2014. “Pheromones, Male Lures, and Trapping of Tephritid Fruit Flies.” In Trapping and the Detection, Control, and Regulation of Tephritid Fruit Flies, 15–74. Springer Netherlands.

[ece372261-bib-0060] Tan, K. H. , I. Tokushima , H. Ono , and R. Nishida . 2010. “Comparison of Phenylpropanoid Volatiles in Male Rectal Pheromone Gland After Methyl Eugenol Consumption, and Molecular Phylogenetic Relationship of Four Global Pest Fruit Fly Species: *Bactrocera Invadens*, *B. dorsalis* , *B. correcta* and *B. zonata* .” Chemoecology 21: 25–33.

[ece372261-bib-0061] Vargas, R. I. , S. K. Souder , B. Mackey , P. Cook , J. G. Morse , and J. D. Stark . 2012. “Field Trials of Solid Triple Lure (Trimedlure, Methyl Eugenol, Raspberry Ketone, and DDVP) Dispensers for Detection and Male Annihilation of *Ceratitis capitata* , *Bactrocera Dorsalis*, and *Bactrocera cucurbitae* (Diptera: Tephritidae) in Hawaii.” Journal of Economic Entomology 105, no. 5: 1557–1565. 23156150 10.1603/ec12122

[ece372261-bib-0062] Verschut, T. A. , K. Farnier , J. P. Cunningham , and M. A. Carlsson . 2018. “Behavioral and Physiological Evidence for Palp Detection of the Male‐Specific Attractant Cuelure in the Queensland Fruit Fly ( *Bactrocera tryoni* ).” Frontiers in Physiology 9: 990. 10.3389/fphys.2018.00990. 30140234 PMC6094961

[ece372261-bib-0063] Virgilio, M. , K. Jordaens , C. Verwimp , I. M. White , and M. De Meyer . 2015. “Higher Phylogeny of Frugivorous Flies (Diptera, Tephritidae, Dacini): Localised Partition Conflicts and a Novel Generic Classification.” Molecular Phylogenetics and Evolution 85: 171–179. 10.1016/j.ympev.2015.01.007. 25681676

[ece372261-bib-0064] White, I. , and M. Elson‐Harris . 1992. Fruit Flies of Economic Significance. CABI. 10.1079/9780851987903.0000.

[ece372261-bib-0065] Wickham, H. , M. Averick , J. Bryan , et al. 2019. “Welcome to the Tidyverse.” Journal of Open Source Software 4: 1686. 10.21105/joss.01686.

[ece372261-bib-0066] Yu, G. , D. K. Smith , H. Zhu , Y. Guan , and T. T. Lam . 2017. “Ggtree: An r Package for Visualization and Annotation of Phylogenetic Trees With Their Covariates and Other Associated Data.” Methods in Ecology and Evolution 8: 28–36. 10.1111/2041-210x.12628.

[ece372261-bib-0068] Zhang, G.‐N. , H. Hull‐Sanders , F. Hu , W. Dou , J.‐Z. Niu , and J.‐J. Wang . 2011. “Morphological Characterization and Distribution of Sensilla on Maxillary Palpi of Six *Bactrocera* Fruit Flies (Diptera: Tephritidae).” Florida Entomologist 94: 379–388. 10.1653/024.094.0301.

[ece372261-bib-0069] Zhang, H. , M. T. Fletcher , J. W. Avery , and W. Kitching . 1997. “A Suite of Odd and Even Carbon‐Numbered Spiroacetals in *Bactrocera Latifrons*. Synthesis and Stereochemistry.” Tetrahedron Letters 38: 3477–3478. 10.1016/s0040-4039(97)00660-6.

[ece372261-bib-0070] Zhang, Y. , H. Li , S. Feng , et al. 2023. “Mitochondrial Phylogenomics Reveals the Evolutionary and Biogeographical History of Fruit Flies (Diptera: Tephritidae).” Entomologia Generalis 43: 359–368. 10.1127/entomologia/2022/1594.

