# Supplementary material for: Divergent Evolutionary Pressures Shape Olfactory Sensitivity of the Maxillary Palps in Tephritidae Fruit Flies
Source: Ecol Evol. 2025 Oct 14;15(10):e72261. doi: 10.1002/ece3.72261 (PMC12519527; doi:10.1002/ece3.72261)
Supplement: Supplementary file 1 — Table S1: Chemical compounds of the three blends (10 ng) of VOCs (40 compounds) used for electrophysiological recordings on maxillary palps of males and females of Bactrocera dorsalis , Bactrocera latifrons , Zeugodacus cucurbitae, Ceratitis capitata and Ceratitis cosyra . The blends were constructed based on the Kovats indices of the synthetic compounds to avoid overlap during elution. [file ECE3-15-e72261-s001.docx]

**Appendix**

Table 1: Chemical compounds of the three blends (10 ng) of VOCs (40 compounds) used for electrophysiological recordings on maxillary palps of males and females of *Bactrocera dorsalis*, *Bactrocera latifrons*, *Zeugodacus cucurbitae*, *Ceratitis capitata* and *Ceratitis cosyra*. The blends were constructed based on the Kovats indices of the synthetic compounds to avoid overlap during elution.

| **Blend** | **Class** | **Compounds** | **CAS number** | **Kovats index** |
| --- | --- | --- | --- | --- |
| *Blend 1* | **Alcohols** | 1-Octen-3-ol | 3391-86-4 | 1433 |
|  | **Esters** | Acetic acid, butyl ester | 123-86-4 | 1061.4 |
|  |  | 1-Butanol, 3-methyl- acetate | 123-92-2 | 1112.2 |
|  |  | Hexanoic acid, methyl ester | 106-70-7 | 1177.4 |
|  |  | Hexyl acetate | 142-92-7 | 1263.5 |
|  |  | 2-Phenethyl propionate | 122-70-3 | 1875.5 |
|  | **Hydrocarbons** | (*E*)-4,8-Dimethyl-1,3,7-nonatriene | 19945-61-0 | 1298.2 |
|  | **Ketones** | Raspberry ketone | 122-48-5 | 2970 |
|  | **Phenols** | o-Cresol | 95-48-7 | 1973.5 |
|  |  | m-Cresol | 108-39-4 | 2061.8 |
|  | **Pyrazines** | Pyrazine, 2,5-dimethyl- | 123-32-0 | 1314.3 |
|  | **Spiroacetals** | Spiros A |  | 1352.4 |
|  |  | Spiros B |  | 1451 |
|  |  | Spiros F |  | 1514.4 |
|  | **Terpenoids** | Caryophyllene | 87-44-5 | 1610.6 |
|  | **Shikimates and Phenylpropanoids** | Zingerone | 5471-51-2 | 2505.4 |
| *Blend 2* | **Esters** | Ethyl caproate | 123-66-0 | 1225.7 |
|  |  | α-Terpinyl acetate | 80-26-2 | 1694.5 |
|  |  | Butanoic acid, 3-methylbutyl ester | 106-27-4 | 1259 |
|  | **Oxygenated hydrocarbons** | 2-Heptanone | 110-43-0 | 1173 |
|  | **Phenols** | 4-Ethylguaiacol | 2785-89-9 | 2012 |
|  | **Spiroacetals** | Olean | 180-84-7 | 1405.5 |
|  |  | Spiros E |  | 1373.8 |
|  | **Terpenoids** | (+)-Limonene | 5989-27-5 | 1195.2 |
|  |  | α- Terpinolen | 586-62-9 |  |
|  |  | Trimedlure | 111790-62-6 | 1797 |
| *Blend 3* | **Esters** | 2-Methylpropyl 3-Methylbutanoate | 589-59-3 | 1184.3 |
|  |  | Acetic acid, pentyl ester | 628-63-7 | 1163.5 |
|  |  | 2-Phenethyl acetate | 103-45-7 | 1805.5 |
|  | **Oxygenated hydrocarbons** | 3-Hydroxy-2-butanone | 513-86-0 | 1261 |
|  | **Phenols** | Phenol | 108-95-2 | 1976.1 |
|  |  | p-Cresol | 106-44-5 | 2053.7 |
|  | **Pyrazines** | 2,3-Dimethyl-pyrazine | 5910-89-4 | 1338.6 |
|  | **Spiroacetals** | Spiros C |  | 1293.2 |
|  |  | Spiros D |  | 1453.5 |
|  |  | Spiros G |  | 1539.4 |
|  | **Shikimates and Phenylpropanoids** | Methyl eugenol | 93-15-2 | 1990.4 |
|  |  | Cuelure | 3572-06-3 | 2567.6 |
|  | **Terpenoids** | trans-β-Ocimene | 3779-61-1 | 1224.8 |
|  |  | Isophorone | 78-59-1 | 1597.8 |


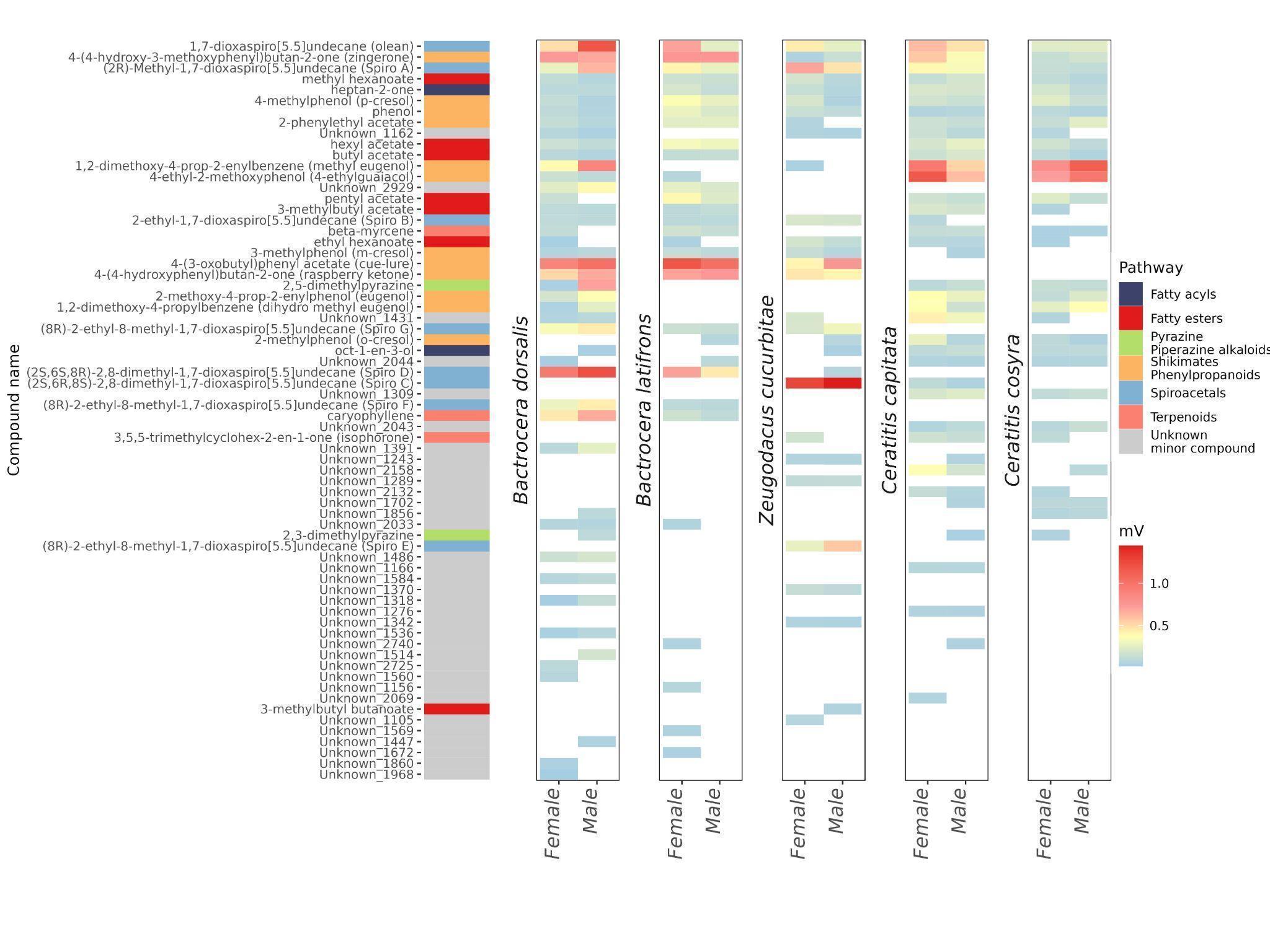


Appendix Figure 1. Heatmap of maxillary palps olfactory sensitivities of *B. dorsalis*, *B. latifrons*, *Z. cucurbitae*, *C. capitata* and *C. cosyra* species to synthetic compounds and impurities whose identification was not resolved . From left to right: a) synthetic compounds and impurities of the three blends, b) their functional class, c) olfactory sensitivities of each species to chemical compounds d) chemical groups for compounds classification, e) the normalized sensitivity of the flies responses ranging from light blue (0) to red (> 1 mv). The compounds are sorted from top to bottom in decreasing order of sharedness across tephritid species and within each cluster of males and females of a single species.
